# Impact of Patients’ Communication with the Medical Practitioners, on Their Adherence Declared to Preventive Behaviours, Five Years after a Coronary Angiography, in Luxembourg

**DOI:** 10.1371/journal.pone.0157321

**Published:** 2016-06-29

**Authors:** Michèle Baumann, Anastase Tchicaya, Nathalie Lorentz, Etienne Le Bihan

**Affiliations:** 1 Research Unit INSIDE, Institute Health & Behaviour, University of Luxembourg, Esch-sur-Alzette, Luxembourg; 2 Luxembourg Institute of Socio-Economic Research (LISER), Esch-sur-Alzette, Luxembourg; Medical University Innsbruck, AUSTRIA

## Abstract

**Background:**

Patients of the National Institute of Cardiac Surgery and Interventional Cardiology in Luxembourg who underwent coronary angiography were surveyed for hypertension, hypercholesterolemia, diabetes and overweight/obesity between 2008/9 and 2013/4. For each cardiovascular risk factor (CVRFs), we analysed the associations between the quality of the patients' communication with the medical practitioner and their adherence declared to preventive behaviours.

**Methods:**

1,289 completed a self-administered questionnaire on communication with the medical practitioner (P’Com-5 items scale; Cronbach 0.87). 61.8% stopped smoking, 57.9% reduced or stopped their consumption of salt, 71.9% of fat, and 62.8% of sugar, and whereas 65% increased their consumption of fruit and vegetables and 19.8% increased their physical activity. Around 37% reported having made changes following their doctor's advice. 90% were followed by a cardiologist and 95.9% by an attending physician.

**Results:**

No link was observed between declaration of physical activity, smoking, fats, and quality of communication. Significant associations: for increased consumption of fruit and vegetables was linked with the quality of doctor-patient communication when patients were overweight (OR = 1.081), obese (OR = 1.130), hypercholesterolemic (OR = 1.102), hypertensive (OR = 1.084) or diabetic (OR = 1.103). Reduction in salt intake was linked only to patients with hypertension (OR = 1.102), whereas reduction or cessation of sugar consumption was linked to overweight (OR = 1.093), and more so obese, (OR = 1.106), hypercholesterolemics (OR = 1.103) and diabetics (OR = 1.173).

**Conclusions:**

Good doctor-patient communication was related to nutrition, particularly increased consumption of fresh fruits and vegetables. Accurate perception of CVRFs by both patients and medical practitioners is essential for CV protection. The aim of instructing patients is to encourage them to make informed decisions about how to change their lifestyle. In routinely, P’Com-5 scale can collect data to assess the improvement of the professional skills. It can be used in medical training to enhance the quality of the therapeutic communication, especially for nutritional coaching, and to evaluate its efficacy in reducing CVRFs.

## Introduction

Evaluation of secondary preventive behavio**u**rs in cardiovascular disease (CVD) is a major public health concern. CVD remains a leading cause of death in European countries and the USA [1, 2]. In the Grand-Duchy of Luxembourg, CVD was the primary cause of mortality and a major reason for hospital admissions in 2012 [3]. Noncompliance to preventative advice is a powerful confounder of evidence-based practice and can affect daily patient management, resulting in inappropriate therapeutic escalation with greater costs and potential for harm. Moreover, it increases the risk of adverse cardiac events, including mortality [4].

Adherence to medical regimens refers to the extent to which patients follow the instructions given to them by practitioners. These instructions include requests for follow-up visits, the taking of medication, and lifestyle changes such as reducing the consumption of sugar, salt and fat, and/or stopping or reducing cigarette smoking [1,5,6,7]. Adherence also involves increasing the consumption of fruits and vegetables, which has a potential protective effect on weight gain and obesity, and reduces plasma cholesterol and the risk of fatal coronary heart disease ^8^. However, although a balanced diet and appropriate level of physical activity lead to education/improvement in cardiovascular risk factors (CVRFs), large numbers of patients fail to comply [1,5,6,7]. Understanding the reasons for noncompliance has become an important area of research. Medical practitioners tend to see noncompliance in terms of uncooperative patients, and underestimate the proportion of their patients who are noncompliant [8]. Patients with chronic heart failure followed by cardiologist described several factors that could inhibit successful communication with their doctors. These included difficulties in the belief that doctors did not want to provide patients with too much knowledge. They find difficult to retain information and may not appreciate the relevance of information provided by clinicians [9].

One plausible explanation for noncompliant behaviour locates the problem within the communication process between medical practitioners and patients–that is, noncompliance results from insufficient or poor communication from physician to patient about the nature of the regimen or a lack of practitioner-patient rapport in general [10]. Doctors and other health professionals play a key role in communicating risk information. They are advisers to patients, especially when patients have to make fateful decisions that can irrevocably change their lives [11]. The Therapeutic Communication Skills of Practitioners scale [12] was developed to assess the quality of doctor-patient relationships and therapeutic communications. Its psychometric properties were validated and the association with the adherence of the patients to their treatments/medication confirmed. Our question here was how adherence to preventive behaviours would be influenced by the quality of the communication between patients and their practitioners. Such associations are not well documented in research into secondary prevention of CVD, but they may be interesting to analyse because they permit us to identify psychosocial and material barriers in an under-researched area.

Improving our understanding of the impact of preventive behaviour on CVRFs is important because awareness of the risk of coronary artery disease can stimulate individuals to change their lifestyles and reduce their chances of having a myocardial infarction or stroke [5]. A telephone-delivered secondary prevention programme using health coaching (ProActive Heart) and developed to improve anxiety outcomes of patients following myocardial infarction, could be effective in improving a range of key behavioural outcomes [13].

Our research hypotheses are that (1) there is a relationship between the quality of communication with the medical practitioner and an increase in patient behaviours that would reduce risk factors. (2) If patients do not display risk factors, doctors do not ask them to modify one or more behavioural patterns. If we take hypertension and salt intake as examples, practitioners have no a priori reason to advise their patients to reduce their salt consumption, if they do not have hypertension; no link between the quality of the communication with the practitioner as perceived by the patient, and salt intake reduction should then be observed.

Greater knowledge of this issue may be useful in improving procedures used to follow up CVD patients at home by allowing adaptation of professional coaching with mobile devices, and improving medical training in therapeutic communication between practitioners and patients who have one or more CVRF. This research created an opportunity to obtain valuable information by monitoring the quality of doctor-patient communication and by improving understanding of the emergence of resistance towards the reduction of cardiovascular risk factors. For each CVRF (hypertension, hypercholesterolemia, diabetes, overweight/obesity), the aim was to analyse the associations between the quality of patient communication with the medical practitioner and adherence declared to preventive behaviours.

## Methods and Material

On the basis of the risk factors observed in 2008/2009, we evaluated the link between the quality of the doctor-patient communication and the evolution of preventive behaviours as measured in 2013/2014.

### Participants and ethical aspects

This survey was a health record audit involving all patients who underwent coronary angiography between 2008 and 2009. Among the 4,391 patients initially contacted, 547 deaths were reported and 209 had an invalid address (moved to institutions, changed their residential arrangements, for example to live with a son or daughter). They were contacted in 2013–2014 by a letter which contained information about the aims of the survey and invited them to complete a self-administered questionnaire in one of three languages: Portuguese, French or German. The protocol was approved by the National Committee of Research Ethics and the Committee for Data Protection of Luxembourg.

### Instrument and data collection

As Luxembourg is multilingual and very culturally diverse (more than 170 different nationalities), our questionnaires were available in the languages of the country. Each version was translated and back-translated, and proofread by native-speaking professional translators.

### Preventive behaviours declared (dependent variables)

Patients were asked if, during the 5 previous years, they had decreased or stopped tobacco consumption, and declared that they increased their physical activity, reduced fat, salt or sugar consumption, and/or increased consumption of vegetables and fruits.

### Communication with the medical practitioner (P’Com-5 items)

In 2013/2014, patients estimated the quality of the relationships with their practitioners on a 10-point scale, five items from a validated scale were selected^10^ (my medical practitioner takes the time to listen to me; gives me incentive to comply with the treatment; gives me advice on prevention (diet, physical activity…); explains to me what the treatment does; gives me information on the side effects of medication). We checked the internal coherence and unidimensionality of the reduced version of the scale. Cronbach's Alpha was 0.870, and the first main factor of the principal component analysis explained 68.3% of the total variance.

- Cardiovascular risk factors (CVRFs) such as diabetes, hypertension, and hypercholesterolemia, and weight and height were self-reported in 2008/9. Based on the International Obesity Task Force, convened by the World Health Organization, a subject with a Body Mass Index (BMI)≥ 30.0 kg/m2 is defined as obese, 25.0–29.9 kg/m² as overweight and <25.0 kg/m² as normal.
- Socioeconomic characteristics: age, sex, whether living in a couple and retirement status were noted.

### Statistical analyses

The P’ Com-5 items score was computed as the average of the responses to the five items of the scale, ranging from 1 (very poor communication) to 10 (very satisfying communication).

For each preventive behaviour declared, we related the probability of improvement since 2008/9 (with regard to the prevention of cardiovascular diseases) and the quality of the doctor-patient communication using a logistic regression model. This relationship was evaluated by the odds ratio; an odds ratio greater than 1 is an indication of a positive relationship between the quality of the doctor-patient communication and the probability of improved behaviour. We introduced an interaction between the quality of communication and each risk factor in order to calculate two distinct odds-ratios, depending on whether individuals had the risk factor in 2008 or not. We assumed that when individuals did not have the risk factor, there was no relationship between the quality of medical practitioner-patient communication and improved behaviour, as patients did not need to improve their behaviour to limit a risk factor they did not have. In this case, we expected to obtain an odds ratio that was not significantly different from 1. Later, we conducted the same analyses adjusting for age and sex, and simultaneously introducing an interaction with the quality of practitioner-patient communication, all the risk factors that could be affected by the introduction of preventive behaviours. It should be mentioned that the communication score has not been dichotomized, but it was introduced in the models in the form of a continuous variable. In the case, the odds ratio should be interpreted as the increase (or decrease) of the logit of the probability of adopting a preventive behaviour corresponding to the increase of the communication score by a unit. Depending on their nature, variables were described using means, standard deviations or percentages. All analyses were performed with SAS 9.4 statistical software (SAS Institute Inc, USA).

## Results

We received 1,289 completed questionnaires, giving an estimated response rate of 35.5% (1,289 / 3,635).

### Socioeconomic characteristics

With regard to the socioeconomic characteristics of the study population, Table 1 describes that the mean age was 69.2 years, 71.4% were men, 74% lived in a couple and about 78.1% of all patients were retired.

**Table 1:** Sociodemographic characteristics of the sample.

		mean (SD) or %
Age		69.2 (11.1)
Sex	Women	28.7
	Men	71.4
Marital status	Living in couple	74.0
Occupation	Retired	78.1

Table 2 shows that in 2008/9, the more common CVRFs were hypercholesterolemia and hypertension (63.7% and 62.8%, respectively). About a quarter of the patients had diabetes and a third had a BMI higher than or equal to 30.

**Table 2:** CV Risk Factors in 2008, healthcare in 2013 and preventive behaviours between 2008 and 2013.

	%
**CV RFs in 2008**	
Hypertension	62.8
Hypercholesterolemia	63.7
Overweight	44.4
Obesity	30.4
Diabetes	24.7
**Healthcare in 2013**	
Hospitalized in the past 5 years for heart conditions	36.6
Followed by a cardiologist	90.0
Followed by an attending physician	95.9
**Over the last 5 years, the patients reported:**	
Increased physical activity^1^	19.8
Smoking cessation^2^	61.8
Reduction in tobacco consumption^2^	19.4
**Patients declared having followed the GP’s advice in regard to:**	
Smoking cessation	35.0
Increased physical activity	37.6
Change of eating habits	38.5
**Over the last 5 years, the patients reported having:**	
Reduced or stopped consuming salt	57.9
Reduced or stopped consuming fats	71.9
Reduced or stopped consuming sugar	62.8
Increased consumption of fruit and vegetables	65.0

^1^ Among those who have no health-related impediments in performing physical activities (83.4% of the total sample).

^2^ Among those who smoked in 2008/2009 (26.0% of the total sample).

Between 2008/9 and 2013/14, 36.6% of patients were hospitalized at least once, 90% were followed by a cardiologist and 95.9% by an attending physician.

Excluding patients who did no physical activity because of their health status, 19.8% declared they increased their physical activity, among whom 37.6% reported having made the change because their doctor had advised them to. Among the smokers, 19.4% reduced their cigarette consumption. The rate of stopping smoking was 61.8%, and 35% of them reported stopping on the recommendation of their doctor.

More than half of the patients reported a favourable change in their eating habits: 57.9% reduced or stopped the consumption of salt, 71.9% of fat, 62.8% of sugar and 65% increased their consumption of fruits and vegetables. Among those who changed their eating habits, 38.5% did so based on the advice of their doctor.

### Associations between medical practitioner-patient communication and preventive behaviours

Table 3 presents that globally, few significant associations were observed, the results of multiple logistic regression models adjusted for age and sex are very close to those of simple logistic models. For simplicity, we will focus on the results of the multiple logistic regressions (right-hand columns).

**Table 3:** Adherence declared of preventive behaviours for each CVRF, and its associations with practitioner-patient communication.

		Multiple logistic regressions					
		(adjusted by age and sex)					
Between 2008 et 2013, evolution of preventive behaviours (dependent variables)	Cardiovascular risk factors	OR	SE	p	OR	SE	p
Decreased salt intake	Hypertension	1.102	0.036	0.003**	1.102	0.036	0.003**
	No hypertension	1.027	0.034	0.422	1.027	0.034	0.422
Reduction or cessation of sugar consumption	BMI: Normal	0.985	0.033	0.656	1.050	0.039	0.190
	Overweight	1.035	0.034	0.292	1.093	0.038	0.012*
	Obesity	1.077	0.036	0.027*	1.106	0.040	0.005**
Reduction or cessation of sugar consumption	hypercholesterolemia	1.066	0.034	0.047*	1.103	0.038	0.005**
	No hypercholesterolemia	1.013	0.033	0.687	1.062	0.037	0.085
Reduction or cessation of sugar consumption	Diabetes	1.177	0.044	< .0001***	1.173	0.045	< .0001***
	No diabetes	1.000	0.032	0.990	0.999	0.033	0.965
Increased consumption of fruit and vegetables	BMI: Normal	1.043	0.034	0.205	1.055	0.037	0.125
	Overweight	1.072	0.034	0.028*	1.081	0.036	0.020*
	Obesity	1.121	0.037	0.001***	1.130	0.039	0.000***
Increased consumption of fruit and vegetables	hypercholesterolemia	1.092	0.035	0.005**	1.102	0.036	0.003**
	No hypercholesterolemia	1.064	0.035	0.054	1.075	0.036	0.032*
Increased consumption of fruit and vegetables	Hypertension	1.086	0.034	0.008**	1.084	0.036	0.014*
	No hypertension	1.081	0.034	0.015*	1.092	0.037	0.009**
Increased consumption of fruit and vegetables	Diabetes	1.109	0.037	0.002**	1.103	0.039	0.005**
	No diabetes	1.071	0.033	0.027**	1.074	0.035	0.026*

Significant p-value

*p < 0.05

**p < 0.01

***p < 0.001.

No link was observed between declaration of increased physical activity and quality of practitioner-patient communication, regardless of the risk factors associated with the presence or absence of physical activity in 2008/9. The same was true for cessation or reduction of smoking and intake of fats. These results were removed from Table 3 in order to facilitate presentation.

Significant associations were all related to food. Declaration of reduction in salt intake was associated with the quality of doctor-patient communication for patients with hypertension (OR = 1.102). Reduction or cessation of sugar consumption was associated with the quality of practitioner-patient communication when the patient was overweight (OR = 1.093), and more so when obese (OR = 1.106). The same held true for patients with hypercholesterolemia (OR = 1.103) or diabetes (OR = 1.173). The same type of association was observed with increased consumption of fruit and vegetables, since its link with the quality of doctor-patient communication was significant in cases of overweight (OR = 1.081), obesity (OR = 1.130), hypercholesterolemia (OR = 1.102), hypertension (OR = 1.084) and diabetes (OR = 1.103).

However, a positive link existed between increased consumption of fruit and vegetables and GP’ Com when patients had no hypercholesterolemia (OR = 1.075), no hypertension (OR = 1.092) and no diabetes (OR = 1.074). We noticed that the effects were stronger when the risk factor was present than when it was not, except for hypertension, in which the results were very close. Except for the increased consumption of fruit and vegetables, no link between the quality of communication and the improvement of preventive behaviours was significant when the risk factors associated with the behaviours were not present.

## Discussion

### Key results

According to the risk factors observed in 2008/9, we analysed the relations of the P’Com-5 items assessing the level of professional competences of practitioners with regard to therapeutic communication perceived by patients, and its effects, five years after a coronary angiography, on adherence declared to preventive behaviours. A notable finding was that good doctor-patient communication was related to nutrition, particularly increased consumption of fresh fruits and vegetables or reduced sugar or fat intake in diabetes, hypercholesterolemia, hypertension, overweight and obesity. Most medical practitioners believe that they provide sufficient information and rationale for patients to fully comply with their instructions and see noncompliance as an irrational response centred on the patient [8]. Physical activity, stopping smoking and reduction of fats are not associated with communication. We suggest that the absence of these relationships results from secondary preventive behaviours which the masses have associated to health for a long time. It is therefore probable that advice from the doctors intervene the least when it comes to these behaviours.

Also, ‘five fresh fruits and vegetables a day’ is a very widely used preventive message, which probably explains the lack of a major difference between people with and without a CVRF. Nutrition is an important determinant of health. Inadequate consumption of fruit and vegetables is one factor that can play a role in increased morbidity [7]. It is necessary to emphasise that our understanding of the potential cardioprotective properties of nutrition is relatively recent. An important evolution in nutritional intervention took place between 1957 and 2013, with a focus on vegetables, fruit, fish, whole grains, and olive oil, and a nuanced recommendation for fat intake [14].

Between 2008/9 and 2013/14, more than three-fifths of our study patients stopped smoking, and stopped or reduced their consumption of salt, fat, and sugar. Consumption of fruits and vegetables increased but only one-fifth declared increased their physical activity. Among them, about two-fifths reported having made the changes based on their doctor’s advice. Maximising the adherence of patients to their treatments/medication and/or the adoption of healthy behaviours, in particular when they return to living at home with their partner and/or family is a real challenge for secondary prevention. They must reorganise their daily lives, adapt to preventive behaviours, adjust to their new lifestyles and available income, as well as rethink their plans for the future. Adherence is more likely when medical practitioners give more explicit and complete instructions and make more decisions with, rather than for, patients [10, 12].

In our study population, more than 9/10 patients regularly visited a cardiologist and an attending physician; the role of the doctor or the specialist who follows the patient is becoming crucial to improving adherence to treatment or secondary prevention behaviours. The emphasis in practitioner-patient relationships is increasingly on patient and doctor sharing responsibility for decision-making and treatment outcomes. This reflects expectations among certain chronic patients for participation in their care, and the increasing proportion of them requiring long-term treatment and lifestyle intervention [15]. Meeting those expectations could markedly improve the therapeutic process, but the question arises of whether practitioners are ready to agree that there is a need to reconsider and modify the care they provide for their patients.

### Added value and generalizability

Five years on, increased consumption of fresh fruits and vegetables was associated with the quality of doctor-patient communication when patients were overweight, more so in cases of obesity and hypercholesterolemia. Crossing the level of intention to modify their behaviour and subsequent practice, researchers [16, 17] attempted to account for the gap between knowledge of CVRFs and the adoption of healthy behaviours. In regard to this perspective, our findings suggested that two profiles of patients would link with the quality of communication: those who are ‘inclined actors’ [16, 17] motivated to change their preventive behaviours and act to do so, and ‘disinclined actors’ [16, 17] who are unwilling, but act anyway. In addition, patients whose intentions to change are more aligned with the moral norms of the society or their community are more likely to adopt preventive behaviours than participants whose intentions differed [18]. Under such circumstances, in the relationships doctor-patient with a chronic disease, some models were proposed [15]. The mutual participation model, the patient’s own experience is an essential component of the therapeutic approach, as he or she is responsible for much of its implementation. In essence, the practitioner helps the patient help him/herself. The negotiated order model between patient and practitioner, whereby the relationship is not predetermined, but established by negotiation and open to modification at any time—for example if the patient requires more information [15]. However, responding to this need is not sufficiently. It does not take into account the complexity of the clinical practice, nor the importance of considering patients as active partners in communication [11].

Between 2008/9 and 2013/14, the same held true for decreased sugar consumption in cases of overweight, obesity, diabetes and hypercholesterolemia, and salt consumption in hypertension. Our findings present the impact of the quality of doctor-patient communication on adherence to diet when the patient has a CVRF. Early adherence is required to arrest or slow down the progression of CVD. Such a strategy could contribute to improving patient understanding of CVRFs, and to motivate, patients, in particular those who have behavioural risk factors to change their lifestyle and adopt healthy behaviours. Accurate perception of CVRFs by both patients and medical practitioners is essential for CV protection. The aim of instructing patients is to encourage them to make informed decisions about how to reduce those CVRFs. The principal reason is to adhere to adequate lifestyle changes. The relationships that patients develop with their medical practitioners are an important determinant, in particular when a substantial proportion of patients have their life satisfaction affected. Satisfaction with life is related to positive mental health outcomes and people who are satisfied with their lives report lower levels of distress [19]. A recent study with the same population demonstrated that patients with a five year history of physical inactivity, angina pectoris, obesity, diabetes, or hypercholesterolemia, were more likely to have lower life satisfaction [20]. In this context, the P’Com-5 items scale appeared to be an appropriate tool for patients who attend cardiac rehabilitation programs in order to improve their knowledge in healthy behaviours.

### Limitations and further research

The strengths and the limitations of the survey reside in four points. First, the small size of the country made it possible to organize data collection at a national level. Our participation rate of 35.5% is similar to that of a previous study (32.2%) in Luxembourg [3]. Second, such study protocols are rare because they are very expensive and difficult to organise. Third, measurement of adherence is a delicate process. Indeed, the diversity of approaches to measurement is the main obstacle to research in this area. The method adopted here—subject report—is the most widely used. It is indirect and often presented in the literature as a simple, reliable tool that is easy to implement. Here we measure the evolution declared of consumption as the effect of adherence to secondary preventive behaviours. The most common criticism is that subjects tend to overestimate their degree of adherence, either to please the interviewer or because they are afraid of his or her disapproval. Fourth, the data, in particular on obesity status, was based on self-reported height and weight. It has been shown that obese people tend to under-report their weight while most people over-report their height [21].

Five years after a coronary angiography, adherence with preventive behaviours, especially regarding nutrition, relies heavily on the practitioners’ advice through appropriate communication. In routinely, the P’Com-5 scale which represents a component of the interpersonal doctor-patient relationship can collect data to assess the improvement of the professional skills that address the needs of healthcare system users. It can also used in medical training to enhance the quality of therapeutic communication and to evaluate its efficacy in reducing CVRF.

Adherence to healthy behaviours, in particular for nutritional coaching, of the CVD patients requires from the practitioner an ability both to listen and to impart relevant information. Effective and better ways of communicating with patients with chronic heart failure need to be tested. Strategies to help patients ask questions, including those related to prognosis, should be developed. In parallel, for patients at home the procedures used to follow up can be facilitated by nutritional coaching using connected of things, and routinely collecting data to assess the impact of prevention adherence declared.
